# Person-organization fit and turnover intention: the mediating role of meaning in work and moderating effect of independence ethical climate among polish employees

**DOI:** 10.3389/fpsyg.2026.1757699

**Published:** 2026-01-29

**Authors:** Marcin Wnuk, Agata Chudzicka-Czupała

**Affiliations:** 1 Department of Work and Organisational Psychology, Faculty of Psychology and Cognitive Science, Adam Mickiewicz University in Poznań, Poznań, Poland; 2 Interdysciplinary Center for Social Activity and Well-Being Research, Faculty of Psychology, SWPS University, Katowice, Poland

**Keywords:** independence ethical climate, job demands-resources theory, meaning in work, person-organization fit, polish employees, turnover intentions

## Abstract

**Objective:**

Based on the job demands–resources (JD-R) theory and the person–organization (P–O) fit concept, this paper verified whether employees’ perception of the congruency of their values with those of their organization is directly and indirectly related to their turnover intentions through meaning in work. Additionally, this paper investigated whether an ethical climate of independence moderates the link between P–O fit and meaning in work.

**Method:**

This cross-sectional study was conducted among 1,071 employees of companies of different sizes in Poland. Participants completed validated measures assessing P-O fit, five types of ethical climate (independence, caring, instrumental, law and code, and rules), meaning in work, and turnover intention. A moderated mediation model was tested using Hayes’ PROCESS macro (Model 7), controlling for demographic and organizational variables. Confirmatory factor analysis established discriminant validity of the constructs.

**Results:**

The study confirmed direct and indirect mechanisms linking P-O fit to turnover intention. P-O fit was negatively related to turnover intention both directly (*b* = −0.26, *p* < 0.001) and indirectly through meaning in work. Crucially, independence ethical climate significantly moderated the P-O fit to meaning in work relationship, such that the positive effect of value congruence on meaningful work was stronger when employees perceived greater freedom to base ethical decisions on their personal moral standards. The moderated mediation index was statistically significant, confirming that the indirect effect of P-O fit on turnover intention through meaning in work varied across levels of independence ethical climate.

**Conclusion:**

P-O fit facilitates meaning in work and protects employees from motivation to quit the organization, particularly when organizations cultivate independence ethical climates. Ethical decisions rooted in employees’ personal moral codes amplify the positive link between P-O fit and meaning in work, which serves as an important antecedent of retention. Practical implications include the importance of values-based recruitment, creating ethical climates that honor individual moral autonomy, and implementing interventions to enhance meaning in work.

## Introduction

With Poland experiencing its lowest unemployment rate in history (Główny Urząd Statystyczny [GUS], 2024), an aging population leading to fewer individuals of working age, and high employee turnover especially among the youngest employees (Kiełczewska and Rozbicka, 2023), the role of employers and their representatives in bonding employees to organizations and limiting their turnover intention is becoming increasingly important. The Polish labor market has undergone dramatic transformation since its EU accession in 2004, with unemployment dropping from 19.9% in 2004 to approximately 5% in 2024, creating unprecedented challenges for talent retention (Eurostat, 2024). This tight labor market, combined with generational shifts in work values, has intensified competition for qualified employees and elevated the strategic importance of understanding factors that promote organizational commitment.

Poland’s historically low unemployment rate reflects multiple interconnected factors. First, demographic shifts including population aging and declining birth rates have reduced the working-age population (Eurostat, 2024). Second, substantial emigration of Polish workers to other EU countries following the 2004 accession has diminished the domestic labor pool (Main and Czerniejewska, 2017). Third, sustained economic growth and increased foreign direct investment have created strong labor demand (OECD, 2024). Fourth, government policies including family support programs and reduced retirement ages for certain groups have further tightened labor supply (Kiełczewska and Rozbicka, 2023). These structural changes have fundamentally transformed Poland from a labor surplus to labor shortage economy, intensifying competition for talent and elevating retention as a strategic priority.

Poland’s cultural context is characterized by high religious homogeneity, with approximately 92% of the population identifying as Catholic (Główny Urząd Statystyczny [GUS], 2022). This religious orientation may influence workplace ethical frameworks and the salience of moral autonomy in organizational settings. Understanding how value congruence and ethical climates operate in this context contributes to knowledge about whether established Western theoretical frameworks generalize to culturally distinct Central European contexts.

This implies the need for companies to implement measures to build lasting relationships with their employees by recruiting individuals who fit their values and culture. Person–organization (P–O) fit is an element of Kristof (1996) wider concept of person–environment (P–E) fit, which refers to the coherence of employees’ individual values, personality traits, goals, and norms with their work environment. This kind of fit between employee and employer is related to positive outcomes, such as organizational commitment (Edwards and Billsberry, 2010), job satisfaction (Andela and van der Doef, 2019; Chen et al., 2023; Edwards and Billsberry, 2010; Lyons et al., 2014; Wnuk, 2017), and work engagement (Krishnan et al., 2023; Memon et al., 2018; Peng et al., 2014), and may prevent harmful organizational phenomena such as burnout (Andela and van der Doef, 2019; Liu and Xie, 2024; Zeng and Hu, 2024), stress (Mostafa, 2016; Waszkowska et al., 2017), and turnover intention (Andela and van der Doef, 2019; Chen et al., 2023; Edwards and Billsberry, 2010; Hoare and Vandenberghe, 2024; Lyons et al., 2014; Saks and Ashforth, 1997). Previous studies have explored the mechanisms underpinning the link between P–O fit and turnover intention by considering the mediating and moderating roles of some variables. For example, studies have confirmed that employee–organization value consistency is indirectly negatively related to motivation to quit the organization, through job satisfaction (Andela and van der Doef, 2019; Chen et al., 2023; Liu et al., 2010) and work engagement (Krishnan et al., 2023; Memon et al., 2018; Peng et al., 2014). According to Wen et al. (2016), the greater the professional identity declared by employees is, the stronger the connection between their P–O fit perception and their turnover intention is. Maden and Kabasakal (2014) found that among employees of Turkish banks, perceived organizational support amplified the negative effect of the perception of value coherence between employees and the organization on motivation to quit the job. Oh et al. (2014) also found that a negative relationship between P–O fit and turnover intention is weaker in a collectivistic national culture and with stronger power distance. However, the psychological mechanisms through which P-O fit influences turnover intention remain incompletely understood. While job satisfaction and work engagement have been established as mediators, the role of meaning in work – a fundamental human need according to positive psychology (Seligman, 2011) – has received limited empirical attention in this relationship. Furthermore, the boundary conditions under which P-O fit more strongly predicts meaningful work remain largely unexplored.

In this study, within the moderated mediation model, the relationship between P–O fit and turnover intention was tested with the mediating function of meaning in work and the moderating impact of an ethical climate of independence. While individual components of this model have been examined in prior research (e.g., P-O fit → turnover intention; autonomy → meaning), their integration within a moderated mediation framework examining these specific mechanisms has not been empirically tested. This model combines positive psychology with the P–O fit theory (Kristof, 1996) and the job demands–resources (JD–R) theory (Bakker and Demerouti, 2017), showing that the congruency between organizational values and employee values facilitates employees’ perception of their work as purposeful and that the impact of this facilitating relationship is stronger when the employees’ ethical decisions are based on their ethical standards. In turn, purposeful work can mitigate employee’s motivation to leave an organization.

While the P-O fit to turnover intention relationship is well-established (Kristof-Brown et al., 2005), critical theoretical gaps remain. First, despite widespread recognition that P-O fit operates through psychological mechanisms, the specific pathways remain incompletely mapped. Most research has examined job satisfaction and organizational commitment as mediators (e.g., Chen et al., 2023; Liu et al., 2010), but these constructs represent evaluative attitudes rather than existential states. Meaning in work—a fundamental psychological need according to self-determination theory and positive psychology (Ryan and Deci, 2017; Seligman, 2011)—represents a qualitatively different mechanism involving identity, purpose, and significance. Yet its mediating role in the P-O fit to turnover relationship remains empirically unexplored.

Second, while research recognizes that contextual factors moderate P-O fit effects (Oh et al., 2014), the specific organizational conditions that amplify or attenuate these effects warrant further investigation. Ethical climate represents a theoretically compelling but empirically understudied boundary condition: it shapes whether and how employees can authentically express values at work. Independence ethical climate specifically addresses moral autonomy—a dimension conceptually linked to both value enactment and meaningful work but never tested as a moderator in the P-O fit process.

Third, most P-O fit research derives from Western, particularly North American, contexts. Evidence from Central European contexts like Poland remains limited, raising questions about cross-cultural generalizability. Poland’s cultural profile (moderate individualism, moderate-high power distance; Country Comparison, 2024) differs from prototypical Western contexts, and its unique labor market conditions (historically tight, aging workforce) create distinct retention dynamics.

This study addresses these gaps by: (1) establishing meaning in work as a novel mediating mechanism grounded in positive psychology and self-determination theory; (2) identifying independence ethical climate as a theoretically meaningful moderator of the first-stage (P-O fit → meaning) relationship; and (3) testing these mechanisms in an underrepresented cultural and economic context. Together, these contributions extend P-O fit theory by specifying when (independence climate) and how (meaning in work) value congruence translates into retention, while demonstrating cross-cultural applicability.

The current study makes several theoretical and practical contributions. We build upon established P-O fit research by identifying meaning in work as an additional mediating mechanism beyond previously studied pathways through job satisfaction and organizational commitment. We extend ethical climate theory by demonstrating that independence climate moderates value congruence effects. Additionally, we contribute cross-cultural evidence by testing these mechanisms in a Central European context, thereby advancing understanding of how value congruence translates into retention outcomes across different cultural settings.

Finally, given Poland’s current demographic and economic challenges, these findings offer actionable guidance for organizations seeking evidence-based retention strategies in competitive talent markets.

## Review of research

### Person–organization (P–O) fit and meaning in work: moderating role of independence ethical climate

P–E fit is one of the most significant theories in organization and work psychology, emphasizing the beneficial role of congruency between employees and their jobs, organizations, supervisors, and team members (Kristof, 1996; Kristof-Brown et al., 2023). P–O fit is an element of P–E fit and encompasses two types of mutual adjustment between employees and organizations: supplementary and complementary (Muchinsky and Monahan, 1987). Supplementary fit arises when employees and organizations share key characteristics, such as values, needs, and goals. Complementary fit occurs when an employee offers something valuable and desirable to an organization that completes the organization, and vice versa.

Person-environment (P-E) fit theory encompasses multiple dimensions of compatibility between individuals and their work contexts. Kristof-Brown and Billsberry (2013) suggest that fit is determined through a systematic evaluation of how well an individual’s attributes align with the characteristics of their environment. Recent research has expanded understanding of how these fit dimensions interact with organizational behaviors and engagement (Armitage and Amar, 2021; Vogel et al., 2020). Within this framework, an independence ethical climate represents a specific environmental dimension that legitimizes the use of personal moral standards in decision-making (Victor and Cullen, 1988; Martin and Cullen, 2006). This climate dimension aligns conceptually with P-O value fit by providing the necessary autonomy for employees to align their internal ethical frameworks with their external professional conduct.

In this study, we focused on the coherence of values and ethical norms between these two parties, whereby the organization provides its employees with values similar to their desired values, which enhances their motivation and potential to meet their needs in the workplace. This study focuses specifically on supplementary P-O fit—the congruence between employee and organizational values—rather than complementary fit. Values represent enduring beliefs about desirable end-states or behaviors that guide decisions and evaluations (Schwartz, 1992). When employees share their organization’s core values (supplementary fit), they experience greater psychological comfort, reduced identity conflict, and enhanced organizational identification (Edwards and Cable, 2009). Values are particularly relevant to meaning in work because meaningful work arises when individuals can enact deeply held values through their occupational activities (Rosso et al., 2010). Thus, value congruence creates foundational conditions for experiencing work as personally significant and aligned with one’s authentic self.

Values are significant factors in finding meaningful work (Rosso et al., 2010). Different generations have distinct values that shape their definition of meaningful work (Weeks and Schaffert, 2019). Traditionalists, baby boomers, and millennials all value altruism, which is reflected in their inclination to help others. The achievement of personal goals is important for both baby boomers and Generation X, but individual satisfaction and happiness are also relevant for both traditionalists and millennials. In addition, traditionalists regard alignment of their organization’s values with their own as important for meaningful work (Weeks and Schaffert, 2019). Indeed, employees whose values are similar to those of their organization can find meaning in their work more effectively. When employees perceive axiological consistency—that is, when they believe their personal values align with organizational values—they experience more authentic work attitudes, enabling them to satisfy fundamental psychological needs for autonomy, competence, and relatedness (Greguras and Diefendorff, 2009; Ryan and Deci, 2017). This alignment can increase their self-efficacy (Wongsuwan and Na-Nan, 2022) and desire to identify with their work as something significant for their identity. These can lead them to see their job as a calling (Oh and Han, 2018). Two studies have considered the connection between P–O fit and meaning in work. In Duffy et al.’s (2015) research, P–E fit—measured by P–O fit, needs–supplies fit, and demands–abilities fit—turned out to be a positive antecedent of meaning in work. Widodo (2024) confirmed that in the Public Works and Public Housing Office of Bengkulu Province, employees’ perception of the congruence of their values with those of their employer positively predicted their finding of meaning in their work. An independent ethical climate may interact with values congruence in explaining meaning in work. An ethical climate lays the foundation for ethical decision-making in an organization (Victor and Cullen, 1988). There are five types of organizational ethical climates. A caring ethical climate focuses on collectivism, altruism, cooperation, and support. An instrumental ethical climate highlights self-interests and individualism. A law and code ethical climate is based on a universal moral codex in a society, such as the Bible. A rules ethical climate is characteristic of and specific to a given organization. Finally, an independent ethical climate is rooted in a set of moral values held by each employee.

Victor and Cullen's (1988) typology identifies five ethical climate types: caring, instrumental, law and code, rules, and independence. While all five types were measured in this study (and included as control variables), we focus theoretically and hypothetically on independence climate for several compelling reasons.

First, independence climate uniquely addresses moral autonomy—the freedom to apply personal ethical standards in organizational decision-making. This dimension aligns most directly with self-determination theory’s emphasis on autonomy as a fundamental psychological need (Deci and Ryan, 1985). Since our theoretical framework proposes that P-O fit facilitates meaning through autonomy satisfaction, independence climate represents the ethical dimension most conceptually relevant to this process.

Second, independence climate specifically concerns value enactment at the ethical-moral level. While P-O fit addresses general value congruence, independence climate operationalizes whether organizations permit employees to authentically apply these values in ethically consequential situations. This creates a theoretically coherent interaction: value congruence (P-O fit) combines with ethical autonomy (independence climate) to enable authentic meaning-making.

Third, other climate types emphasize external referents (laws, organizational rules, collective welfare, and self-interest) rather than personal moral agency. These external ethical frameworks may matter for compliance and coordination but less directly relate to the autonomous value enactment we theorize drives meaningful work.

Fourth, preliminary theoretical considerations and prior research (Ambrose et al., 2008; Sims and Keon, 1997) specifically implicate independence climate in person-ethical fit processes, whereas evidence for other climate types playing similar moderating roles remains limited.

We acknowledge this focused approach trades breadth for theoretical precision. Future research examining whether other ethical climate dimensions interact differently with P-O fit would provide valuable extensions. However, for testing the specific autonomy-based mechanism we propose, independence climate represents the most theoretically appropriate moderator.

Organizations with an independent ethical climate encourage employees to behave according to their own sets of moral standards (Martin and Cullen, 2006). In ambiguous moral situations or moral conflicts, decision-making requires discernment, following internalized norms, rules, principles, and values (Reynolds, 2006; Treviño et al., 2006). In such a climate, P–O ethical value fit significantly influences employee attitudes toward the organization. The studies have confirmed that perception of ethical climate and ethical leadership may impact organizational citizenship behavior (Newman et al., 2014; Sharif and Scandura, 2014). Ambrose et al. (2008) found that consistency between employees’ perceived independent ethical climate and their individual ethical values positively predicted job satisfaction and negatively predicted turnover intention. These findings have been replicated in subsequent research demonstrating that person-ethical climate fit predicts organizational commitment and reduces withdrawal intentions (Cheng et al., 2019; Demirtas and Akdogan, 2015; Köroğlu, et al., 2024). Moreover, in a sample of working students, the difference between their desired independent ethical climate and their actual perception of this climate was positively related to their turnover intention (Sims and Keon, 1997). These findings have been substantiated by recent research demonstrating that misalignment between preferred and perceived ethical climates predicts job satisfaction, and withdrawal cognitions and behaviors across diverse occupational contexts (Mulki et al., 2008; Ryu, 2020).

Analogous results were seen in Sims and Kroeck’s (1994) study, and additionally, the discrepancy between employees’ preferred independent ethical climate and the perception of this climate negatively predicted affective organizational commitment. When employees share their organization’s values, they can find even more meaning and purpose in their work if they can freely make ethical decisions in the workplace rooted in their own moral code.

Our model proposes that independence ethical climate moderates—rather than causes—the relationship between P-O fit and meaning in work. Specifically, employees who experience value congruence with their organization (high P-O fit) and who work in climates that honor individual moral autonomy find even greater meaning in their work because they can authentically enact their values without ethical compromise (Deci and Ryan, 1985, 2020). This alignment between personal values, organizational values, and ethical autonomy creates conditions for optimal self-expression and moral integration at work. The favorable role of work autonomy in finding significance and purpose in work was confirmed by studies on the job characteristics theory (Hackman and Oldham, 1980; Martela et al., 2021), which posits that for a job to be meaningful, it should be designed with the following core characteristics: skill variety, task identity, task significance, feedback, and autonomy. The theory emphasizes the need for autonomy as basic and universal for every human being, as well as crucial in the motivational process. For example, Anthun and Innstrand (2016) found that employees in three group ages who experienced job autonomy found more meaning in work. Albrecht et al. (2021) extended this finding—they discovered that employees who experienced job autonomy found more meaning in work, which enhanced their work engagement. On the other side, the need for autonomy is a consequence of P–O fit (Greguras and Diefendorff, 2009), which suggests that the need for autonomy helps explain the relationship between P–O fit and meaning in work and that this relationship is strengthened when employees perceive an independent ethical climate, which encourages them to make ethical decisions at work autonomously based on their own moral values and standards.

From these findings, we propose the following hypotheses (H):

*H1*: Among Polish employees, the perception of an independent ethical climate moderates the relationship between P–O fit and meaning in work.

*H2*: Among Polish employees, P–O fit is negatively related to turnover intention.

### Meaning in work and turnover intention

The benefits of meaningful work are highlighted in numerous approaches to this topic. In logotherapy (Frankl, 1992), the “will to meaning” is the primary and universal motive for action. It energizes and organizes the relationship between individuals and their surrounding reality, making such relationship predictable, coherent, and manageable. This approach focuses on the propensity of human beings to find purpose in life and on the individual abilities involved in this process, regardless of external influences. In the occupational sphere, this approach suggests that man can find meaning in work despite external obstacles and can overcome them via transcendence. In this context, Frankl can be seen as a precursor of workplace spirituality, focused on finding purpose in work (Singh and Singh, 2022). Some authors connect the psychology of religion and spirituality to positive psychology as a common framework for religious or spiritual meaning systems at work, leading to positive outcomes (Dik and Alayan, 2023).

As mentioned earlier, experiencing meaning in work is central in job characteristics theory (Hackman and Oldham, 1980). Designing jobs characterized by skill variety, task identity, task significance, feedback, and autonomy is key to finding meaning in work (Hackman and Oldham, 1975). Creating these conditions enables employees to experience positive states, such as meaningfulness, responsibility, and knowledge of results, which, in turn, increase motivation, satisfaction, and performance and decrease absenteeism and turnover (Hackman and Oldham, 1975). Previous studies have confirmed the positive role of purposeful work in enhancing job satisfaction (Duarte-Lores et al., 2023; Duffy et al., 2015), employee commitment to the organization (Jung and Yoon, 2016), work engagement (David and Iliescu, 2022; Jung and Yoon, 2016; Paola et al., 2023), and organizational citizenship behaviors (David and Iliescu, 2022; Paola et al., 2023), as well as in mitigating burnout (Krok, 2016), stress (Kuchinke et al., 2010), turnover intention (Leunissen et al., 2018), and absences due to long-term sickness (Clausen et al., 2014).

### Theoretical integration: JD-R framework

Job demands-resources (JD-R) theory (Bakker and Demerouti, 2017, 2014) provides an integrative framework for understanding these relationships. JD-R theory proposes that workplace factors can be classified as either demands (aspects requiring sustained effort and associated with costs) or resources (aspects that facilitate goal achievement, reduce demands, or stimulate personal growth). Resources operate through motivational processes: they fulfill basic psychological needs, foster engagement and positive work states, and promote organizational attachment.

Critically, JD-R theory recognizes personal resources—individuals’ positive self-evaluations and personal characteristics—as functioning similarly to job resources (Xanthopoulou et al., 2007). Person-organization fit represents a personal resource: the psychological asset of value congruence that facilitates organizational identification, reduces identity conflict, and enables authentic self-expression (Edwards and Cable, 2009). This personal resource should foster other positive psychological states, including meaning in work.

Independence ethical climate functions as a job resource by providing autonomy support in the ethical domain. According to JD-R theory, resources often interact: multiple resources can create gain spirals wherein resources mutually reinforce each other (Hobfoll, 2002). Thus, we propose that independence climate (job resource) amplifies the effects of P-O fit (personal resource) on meaning in work through resource synergy.

Meaning in work itself represents both a psychological state and a personal resource. As a positive work state, it reflects fulfilled needs for significance and purpose. As a personal resource, it provides psychological capital that buffers against withdrawal cognitions by anchoring employees’ organizational attachment in existential rather than merely transactional bonds.

Within this framework, our model proposes a resource-driven motivational process: personal resources (P-O fit) combine synergistically with job resources (independence climate) to generate positive psychological states (meaning in work), which in turn reduce withdrawal cognitions (turnover intention) through enhanced organizational attachment. This represents a classic JD-R motivational pathway wherein resources build upon each other to produce positive outcomes.

This study is theoretically rooted in two concepts: P–O fit (Kristof, 1996) and JD–R theory (Bakker and Demerouti, 2017). P–O fit facilitates finding meaning in work, and this positive relationship is strengthened by the coherence of both parties’ ethical standards. According to JD–R theory, coherent values and purposeful work can be considered personal resources, as are optimism and self-efficacy, which can perform the same function as job resources of helping employees to cope effectively with job demands (Bakker and Demerouti, 2017). *Job demands* are physical, psychological, social, or organizational elements of work that require permanent physical and/or psychological involvement and have physiological and/or psychological costs (Schaufeli and Bakker, 2004). Employees’ preference for the same values as those of the organization is a resource that promotes organizational identification (Malhotra et al., 2022) and organizational attachment (Edwards and Billsberry, 2010), which enhance employees’ potential to find purpose in work. This purpose weakens their motivation to leave the organization. This effect is potentially amplified by the congruence of the ethical norms of these two parties, which are the points of reference in ethical decisions and dilemmas. The ability to base one’s ethical decisions at work on one’s own ethical standards is a means of filling one’s need for autonomy, which is important in the process of searching for meaning (Martela et al., 2018).

From these findings, we propose the following hypotheses:

*H3*: Among Polish employees, meaning in work is negatively related to turnover intention.

*H4*: Among Polish employees, meaning in work mediates the relationship between P–O fit and turnover intention, and this mediational effect is moderated by the perception of an independent ethical climate.

## Method

### Procedure

The survey was conducted in Poland. Participants were recruited through multiple channels including social media platforms (LinkedIn, Facebook), professional networks, and direct organizational contacts. They did not receive any financial incentive for participating in this study. The data were collected on an online platform. The study was conducted in accordance with the Declaration of Helsinki and it received ethical approval from the Ethics Committee at the SWPS University, Katowice, Poland (Ethics code: WKEB89/11/2023). The participants were informed about the anonymity of the research and their right to withdraw from the study at any time without giving any reason and without any consequence. Their informed consent was secured online before the data collection was commenced.

To ensure data quality, attention check items were included. The study information clearly described the research purpose, voluntary nature, and confidentiality protections. The survey took approximately 15–20 min to complete.

### Sample

This study included 1,071 adult participants in Poland who were working in various companies in the country. Considering that the research model included 10 predictors, this sample size was sufficiently large, as confirmed by a power analysis. Applying G*Power 3.1.9.7. with linear multiple regression, a fixed model, and R quadrant deviation from 0. A medium effect size of 0.15 with an alpha of 0.05, a power of 0.95, indicated 172 participants as the minimum required sample size. The study employed a non-probability convenience sampling technique, supplemented by purposive sampling criteria. Participants were recruited from the SWPS University research pool using the SONA Systems platform. This method was chosen to efficiently reach a specific population of interest: working students who meet predefined professional experience requirements. All participants who completed the survey met the inclusion criteria, including adulthood (defined as being at least 18 years old), and possessing a minimum of 1 year of overall seniority. These criteria were verified using screening questions at the beginning of the survey. Participants who did not meet these requirements were automatically redirected, and their responses were excluded from the final sample.

A detailed overview of the participants’ demographic and organizational characteristics is provided in Table 1. The demographic profile was predominantly female (80.3%, *n* = 860), with males and other gender identities representing 18.7% (*n* = 200) and 1% (*n* = 11) of the group, respectively. The participants’ average age was *M* = 28.64 years (SD = 8.74), with a mean professional tenure of *M* = 7.88 years (SD = 7.04). Educational attainment was high, with 50.3% holding secondary education and 49.1% possessing higher education degrees. Professionally, the sample was diverse: ordinary workers (40.3%) and specialists (38.5%) formed the majority, while management roles across various levels accounted for the remaining 21.2%. The participants represented various organizational scales, including large (36.1%), micro (26%), small (23.7%), and medium-sized (14.2%) enterprises.

**Table 1:** Sample characteristics (*N* = 1,071).

Variable	*n* (%)
Gender	
Men	422 (18.7)
Women	860 (80.3)
Other	11 (1)
Age (in years)	28.64 (8.74)^a^
Education level	
Primary	2 (0.2)
Vocational	4 (0.4)
High school	539 (50.3)
Higher	526 (49.1)
Seniority (in years)	7.88 (7.04)^a^
Seniority in present work (in years)	3.52 (4.52)^a^
Types of employment	
Contract of employment	528 (49.3)
Mandate contract	448 (41.9)
B2B contract	94 (8.8)
Company size	
1–9 employees	278 (26)
10–49 employees	254 (23.7)
50–249 employees	152 (14.2)
250 employees and more	387 (36.1)
Position held	
Regular employee	432 (40.3)
Specialist	412 (38.5)
Lower-level manager	50 (4.7)
Middle-level manager	106 (9.9)
Higher-level manager	71 (6.6)

^a^For continuous variables means and standard deviations (in parenthesis) are presented.

### Measures

#### Ethical climate

The ethical work climates of the participants’ employer companies were examined using the Polish version of the Ethical Work Climate Questionnaire (Wnuk et al., 2024). This measure verifies employees’ perceptions of their organization’s ethical work climate and categorizes such climate into one of the following types: instrumental, caring, law and code, rules, and independent (Victor and Cullen, 1988). The items were rated on a 5-point Likert scale (from 1 = *strongly disagree* to 5 = *strongly agree*). This tool demonstrated satisfactory psychometric properties (Victor and Cullen, 1988; Wnuk et al., 2024).

#### Meaning in work

Meaning in work was assessed using items related to the dimension of *personal meaning* from the Work and Meaning Inventory (Steger et al., 2012). The original three-dimensional structure of the inventory was not replicated for Polish conditions, as the two-factor solution was a good fit for the data (Puchalska-Kamińska et al., 2019). The *personal meaning* factor encompassed six items rated on a 5-point Likert scale that ranged from 1 (*absolutely untrue*) to 5 (*absolutely true*).

The meaningful work literature encompasses diverse conceptualizations and measurement approaches (Both-Nwabuwe et al., 2017). Scholars distinguish between meaning in work (the subjective experience of significance derived from work activities) and meaningful work (objective characteristics that make work inherently worthwhile). Additionally, measures differ in whether they emphasize the sources of meaning (e.g., task characteristics, social contribution) versus the psychological experience of meaningfulness (Rosso et al., 2010).

The Work and Meaning Inventory (WAMI; Steger et al., 2012) was selected and used for several reasons. First, WAMI focuses on experienced meaningfulness—the subjective sense that one’s work is personally significant—which aligns with our theoretical interest in how value congruence shapes psychological states rather than objective work characteristics. Second, WAMI has demonstrated cross-cultural validity and has been validated in Poland (Puchalska-Kamińska et al., 2019).

However, the Polish validation revealed a two-factor structure (personal meaning and greater good motivation) rather than the original three-factor structure (positive meaning, meaning-making through work, greater good motivations). We utilized only the personal meaning dimension, which captures individuals’ sense that their work is personally significant and aligned with their identity. This dimension most directly reflects the meaning-making process we theorize results from P-O fit and ethical autonomy.

While alternative measures exist (Batuchina et al., 2025; Lips-Wiersma and Wright, 2012), WAMI’s personal meaning dimension most precisely captures the subjective meaningfulness we conceptualize as mediating between value congruence and turnover intention. This measurement decision emphasizes experienced significance over other meaning dimensions (e.g., contribution to others, spiritual fulfillment), which may explain the modest mediation effect observed.

#### Turnover intention

Turnover intention was assessed using a three-item measure (Wnuk, 2017). The participants’ responses were structured a 5-point Likert scale that ranged from 1 (*strongly disagree*) to 5 (*strongly agree*).

#### P–O fit

The congruence of the employees’ values with their organization’s values was verified using the Polish version of the Person–Organization Fit Scale (Wnuk, 2017). Responses to the three questions corresponded to a 5-point Likert scale that ranged from 1 (*I definitely disagree*) to 5 (I definitely agree). Then, the scores were summed up (Cable and DeRue, 2002).

### Data analysis

**Figure 1:**
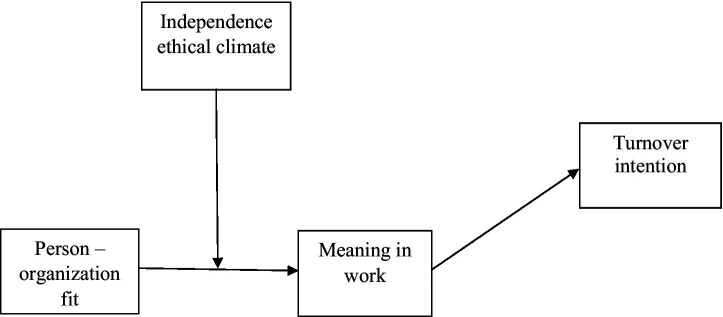
Conceptual model.

The research model is shown in Figure 1: the moderated mediation model, with the P–O fit as the independent variable, meaning in work as the mediating variable, an independent ethical climate as the moderator, and turnover intention as the dependent variable. The following control variables were introduced to the model: sex, age, education, seniority, seniority in the present work, company size, employee position level, a caring ethical climate, an instrumental ethical climate, a law and code ethical climate, and a rules ethical climate.

Multicollinearity was tested using the computed variance inflation factor (VIF).

The confirmatory factor analysis (CFA) was applied to verify discriminant validity of the constructs.

The moderated mediation model was tested in SPSS using Model 7 of the Macro PROCESS prepared by Hayes (2018).

Given the cross-sectional, single-source design, we assessed potential common method variance (CMV) using multiple approaches (Podsakoff et al., 2003). First, procedural remedies were implemented during data collection: we assured respondent anonymity, counterbalanced item order, and used different response formats across constructs where feasible.

Second, we conducted Harman’s single-factor test: a confirmatory factor analysis loading all items onto a single factor yielded poor fit (CMIN/DF = 35.24, RMSEA = 0.179, CFI = 0.75), suggesting that a single common method factor does not account for the majority of covariance.

Third, the significant interaction effect (independence climate × P-O fit) provides evidence against severe CMV bias, as method variance typically attenuates rather than creates interaction effects (Siemsen et al., 2010).

While these assessments suggest CMV does not fully account for our findings, we acknowledge that cross-sectional, self-report designs cannot definitively rule out method bias. Future research employing longitudinal designs, multiple data sources, or experimental manipulations would strengthen causal inferences.

## Results

### Preliminary results

The VIF values of the study variables were less than the threshold of 5 (Table 2; Akinwande et al., 2015), confirming the lack of multicollinearity.

**Table 2:** Descriptive statistics (*n* = 1,071).

Variable	Minimum	Maximum	Mean	Standard deviation	Skewness	Kurtosis	VIF	Cronbach’s alpha coefficient
Person -organization fit	3	15	9.44	3.3	−0.27	−0.56	1.58	0.91
Independence climate	4	20	11.10	3.64	−0.16	−0.36	1.25	0.86
Meaning in work	6	30	19.92	6.50	−0.33	−0.63	1.43	0.93
Turnover intention	3	15	8.31	4	0.18	−1.19	-	0.91

VIF, variance inflation factor.

Confirmatory factor analysis (CFA) using maximum likelihood estimation evaluated discriminant validity by comparing four nested models: (1) one-factor, (2) two-factor combining turnover intention and meaning in work, (3) three-factor combining turnover intention, meaning in work, and P-O fit, and (4) the hypothesized four-factor model. Model fit was evaluated using established guidelines: Chi-square/df (acceptable < 5), RMSEA (acceptable < 0.08), SRMR (acceptable < 0.08), CFI and TLI (acceptable > 0.90). The four-factor model demonstrated superior fit (see Table 3), with all loadings exceeding 0.70 (*p* < 0.001). Discriminant validity was confirmed using the Fornell-Larcker criterion: AVE values (P-O fit = 0.79, independence climate = 0.67, meaning in work = 0.71, turnover intention = 0.78) exceeded squared correlations, with the highest *r*^2^ = 0.29 between P-O fit and meaning in work. The correlation values of the endogenous factors ranged from 0.31 to −0.58, confirming the discriminant validity of the research variables as separate constructs.

**Table 3:** The results of CFA for all measures used in the research measures (*n* = 1,071).

Number of model	Factors	CMIN/DF	RMSEA	SRMR	GFI	CFI	TLI	NFI
One factor model	TI, MW, PO, IC	35.24 (*p* = 0.000)	[0.179, 90% (0.174; 0.184)]	0.1270	0.73	0.75	0.68	0.75
Two factor model	TI + MW, PO, IC	18.39 (*p* = 0.000)	[0.127, 90% (0.122; 0.133)]	0.1024	0.86	0.87	0.83	0.87
Three factor model	TI + MW + PO, IC	11.26 (*p* = 0.000)	[0.098, 90% (0.093; 0.103)]	0.1015	0.91	0.92	0.90	0.92
Four factor model	TI + MW + PO + IC	2.69 (*p* = 0.000)	[0.040, 90% (0.034; 0.46)]	0.0287	0.97	0.98	0.98	0.98

TI, turnover intention; MW, meaning in work; PO, person-organization fit; IC, independence ethical climate.

The correlation coefficients of the study variables are presented in Table 4.

**Table 4:** Values of *r*-Pearson correlation coefficients between research variables (*n* = 1,071).

	P-O Fit	IC	MIW	TI	Seniority	SPW	PH	EL	Age
1. P–O Fit									
2. Independence climate	0.44**								
3. Meaning in work	0.54**	0.32**							
4. Turnover intentions	−0.53**	−0.33**	−0.39**	.					
5. Seniority	0.12**	0.01	0.23**	−0.09**					
6. Seniority in present work	0.06*	−0.04	0.14**	−0.04	0.66**				
7. Position held	0.20**	0.10**	0.25**	−0.18**	0.49**	0.39**			
8. Educational level	0.07*	−0.01	0.16**	−0.02	0.54**	0.35**	0.34**	.	
9. Age	0.09**	−0.04	0.22**	−0.07**	0.93**	0.63**	0.48**	0.63**	
10. Company size	−0.15**	−0.23**	−0.10**	0.15**	0.07*	0.05	−0.01	0.10**	0.09**

*p < 0.05, **p < 0.01. IC – independence climate; MIW – meaning in work; TI – turnover intentions; SPW – seniority in present work; PH – position held; EL – educational level.

P–O fit, independence, ethical climate, and meaning in work were positively intercorrelated and negatively related to turnover intention.

### Hypothesis test results

H1, that an independent ethical climate moderates the relationship between P–O fit and meaning in work, was supported (see Table 5).

**Table 5:** Moderating effects of person-organization fit on meaning in work for different values of independence ethical climate (*n* = 1,071).

Independence climate	Effect	SE	*t*	*p*	LLCI	ULCI
−3.61 (−1 *SD*)	0.694	0.078	8.87	0.0000	0.5406	0.8478
0.00 (*M*)	0.818	0.070	11.63	0.0000	0.6803	0.9565
3.61 (+1 *SD*)	0.943	0.088	10.76	0.0000	0.7706	1.1145

Persona-organization fit as independent variable and meaning in work as outcome variable.

**Figure 2:**
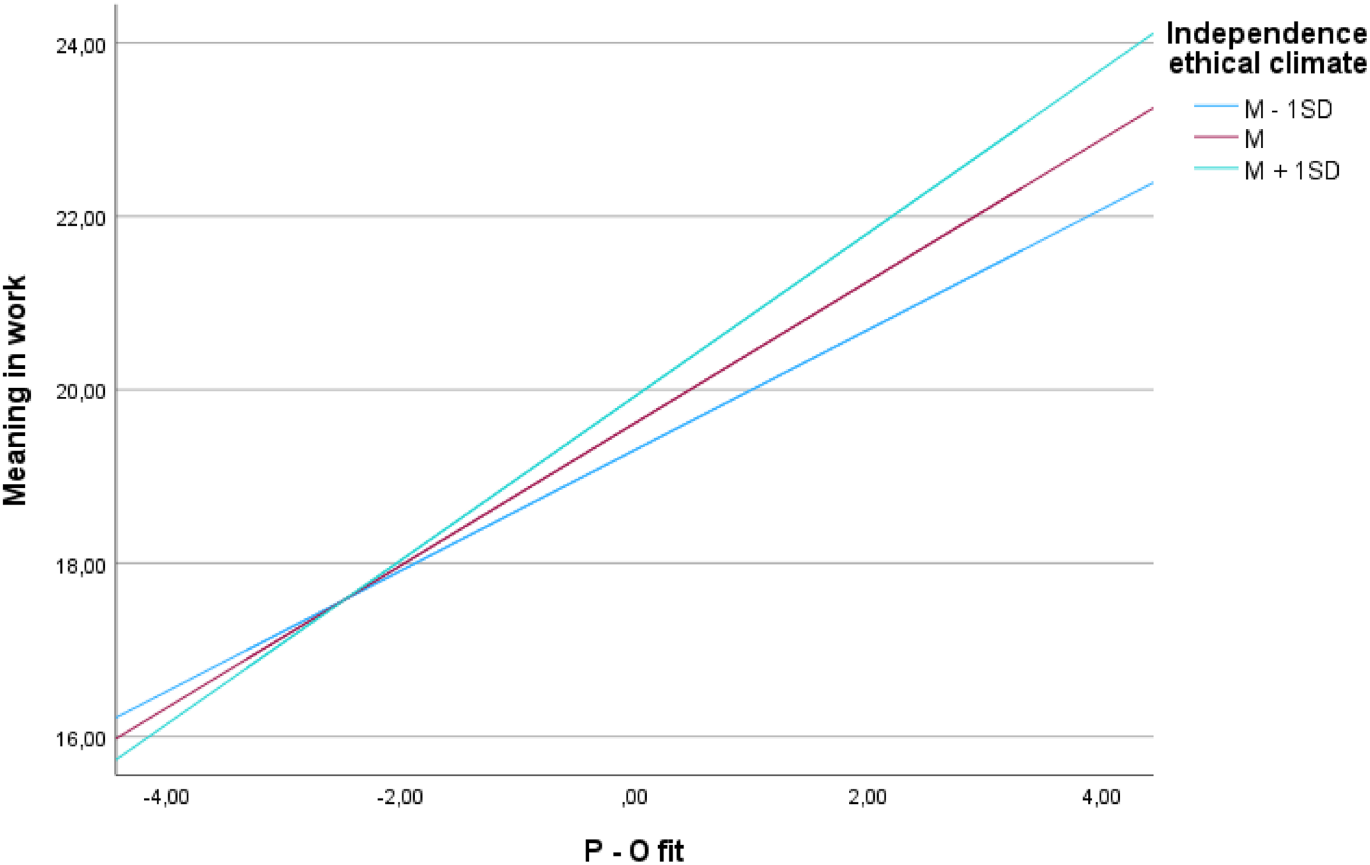
Moderating effect of employees’ perception of independence, ethical climate on the association between P-O fit and turnover intentions.

The moderating effects (Aiken and West, 1991) are shown in Table 5 and illustrated in Figure 2. As expected, the employees’ referral to their internal ethical codex when making ethical decisions in the workplace strengthened the positive effect of P–O fit, as they found meaning in their work.

H2, that P–O fit negatively predicts turnover intention, was confirmed. A negative direct effect of P–O fit on motivation to leave the organization was found (*b* = −0.26; *t* = −5.98, *p* = 0.0000, CI [−0.3474, −0.1757]).

Consistent with H3, that meaning in work is a negative predictor of turnover intention, we observed a statistically significant negative direct effect of meaningful work on intention to leave an organization (*b* = −0.15; *t* = −7.83, *p* = 0.0000, CI [−0.1895, −0.1136]).

H4, that the relationship between P–O fit and turnover intention is mediated by meaning in work and moderated by an independent ethical climate, was also supported. The moderated mediation index was statistically relevant (−0.005, 95% CI [−0.0093, −0.0012]).

Conditional indirect effects analysis revealed that at low independence ethical climate (−1 SD), the indirect effect was −0.105, 95% CI [−0.1495, −0.0683]; at mean levels, −0.124, 95% CI [−0.1676, −0.0849]; and at high levels (+1 SD), −0.143, 95% CI [−0.1913, −0.0974]. As independence climate increases, P-O fit more strongly predicts meaning in work, which more strongly predicts reduced turnover intention, demonstrating that ethical autonomy amplifies value congruence effects on retention through meaning-making processes.

## Discussion

This study tested two mechanisms underpinning the link between P–O fit and the turnover intention of Polish employees: direct and indirect. The indirect mechanism involves the mediating role of meaning in work and the moderating function of an independent ethical climate. The results showed that consistent with previous studies (Andela and van der Doef, 2019; Liu and Xie, 2024; Zeng and Hu, 2024), values congruence between employees and their organization motivates them to stay in the organization.

In the context of P–O fit (Kristof, 1996), similarities between the two parties reduce the probability of the employees searching for reasons and premises for leaving the organization and looking for a new job. Both direct and indirect effects of P–O fit on turnover intention were observed, suggesting the potential of value coherence to mitigate employees’ motivation to quit the organization. Polish employees who perceive their organization’s values as the same as theirs are more likely to finding meaning in their work, and this effect is amplified by the organization’s incentives for employees’ to decide on ethical topics following their ethic codex and norms.

In turn, employees’ conviction about their work is a meaningful antecedent of turnover intention. These findings are consistent with the P–O fit approach (Kristof, 1996) and show that for Polish employees, the congruence of both their values and their ethical norms and rules with those of their organization can boost their search for meaning in work and mitigate their motivation to leave an organization. The findings are also consistent with JD–R theory (Bakker and Demerouti, 2017) and emphasize that alignment of values and ethical standards between employees and their organization, as well as finding meaning in work, are personal resources that can be effectively used to cope with job demands and mitigate turnover intention.

However, the predictive effect of meaning in work on turnover intention was weak in this study but still comparable with that in previous studies (Allan et al., 2019), and it was almost the same as the indirect effect of P–O fit on turnover intention in the group of employees who scored higher than average in independent ethical climate perception. Of all the identified effects, the strongest was the predictive role of P–O fit in turnover intention.

### Theoretical interpretations

The positive relationship between P-O fit and meaning in work can be understood through multiple psychological mechanisms. First, identity-based processes play a central role: when employees perceive that their organization shares their core values, they experience self-concept verification wherein organizational membership affirms their identity (Swann et al., 2007). This identity alignment creates psychological coherence, reducing cognitive dissonance and enabling employees to view their work as an authentic expression of self rather than a compartmentalized role (Cable and Edwards, 2004).

Second, value congruence facilitates autonomy satisfaction by reducing internal conflict between personal convictions and organizational expectations. Employees need not suppress or compromise their values to meet organizational demands, thereby experiencing greater volitional functioning (Ryan and Deci, 2017). This autonomy enables them to find personal significance in work activities because their efforts reflect genuine choice rather than external coercion or compliance.

Independence ethical climate specifically amplifies the P-O fit to meaning relationship through three mechanisms. First, it enhances ethical autonomy by explicitly legitimizing employees’ use of personal moral standards in organizational decision-making (Martin and Cullen, 2006). This autonomy satisfaction at the ethical-moral level reinforces the authenticity derived from value congruence, creating synergistic effects on meaning.

Second, independence climate enables moral agency—employees’ capacity to act as moral agents rather than mere rule-followers (Bandura, 2006). When organizations respect individual ethical judgment, employees experience themselves as responsible moral actors whose work has ethical significance. This moral agency contributes to perceptions of work as meaningful because it involves consequential decisions aligned with personal integrity.

Third, independence climate reduces moral dissonance by minimizing conflicts between personal ethics and organizational ethical expectations. Even when employees share organizational values broadly, specific ethical dilemmas may arise. Independence climate provides psychological permission to resolve these dilemmas according to personal moral codes, maintaining the integrity of the meaning-making process.

The modest indirect effect of meaning in work has several explanations. First, meaning may be more distal from turnover decisions than proximal attitudes like job satisfaction (Tett and Meyer, 1993). Second, Poland’s tight labor market (unemployment ~5%) means employees may leave meaningful work for better external opportunities (Eurostat, 2024). In this context, the strong direct effect of P-O fit (*b* = −0.26) suggests that value alignment serves as a critical differentiator when employees evaluate whether to stay or explore alternatives. Organizations offering authentic value congruence provide something competitors cannot easily replicate, creating psychological bonds that transcend transactional inducements. However, even meaningful work shows modest effects when alternatives abound, indicating organizations must combine value-based strategies with competitive compensation.

Third, methodological factors may contribute. The cross-sectional design may underestimate meaning’s true effect due to reciprocal causation: employees who intend to stay may retrospectively find more meaning in their work, creating bidirectional relationships that attenuate measured effects. Additionally, common method variance may inflate the direct P-O fit to turnover relationship relative to the indirect pathway through meaning (Podsakoff et al., 2012). Fourth, meaning in work may overlap conceptually with other constructs such as work engagement, job involvement, or organizational commitment, creating multicollinearity that reduces unique variance explained. When multiple related positive work states are considered simultaneously, each individual construct may show modest effects.

### Social identity and organizational belonging

This study’s outcomes can be explained within the social identity theory framework (Turner et al., 1979). The alignment of values and ethical standards between employees and their organization facilitated social identification, which, in turn, fostered a sense of belonging, purpose, self-worth, and identity. Instilled feelings of connection and unity, direction and purpose, being part of a larger community, and pride in the organization’s achievements motivated the employees to stay in the organization, as they viewed it as a positive reference group for building their social identity.

More specifically, when employees perceive that their organization shares their core values, they experience enhanced self-concept verification, wherein the organization becomes an extension of their identity. This identity fusion may create psychological bonds that transcend transactional employment relationships, making departure psychologically costly as it would require abandoning a valued aspect of one’s social identity (Swann et al., 2012). The moderating role of independence ethical climate further supports this interpretation-when organizations permit autonomous ethical decision-making aligned with personal moral codes, employees experience authenticity in their organizational membership, strengthening identity-based attachment.

### Self-determination and autonomy

Self-determination theory also proved to be an appropriate base for interpreting the study results and the need to emphasize the role of autonomy in connections between P–O fit, an independent ethical climate, and meaning in work. Functioning in an organization and making decisions motivated by values and ethical norms congruent with one’s own and relevant to one’s self-identity can lead to the conviction that work is meaningful and has a purpose (Deci and Ryan, 1985). Greguras and Diefendorff (2009) showed that P–O fit precedes the satisfaction of the need for autonomy. In turn, a longitudinal study by Martela et al. (2021) confirmed that the need for autonomy drives the search for significance in work.

The current findings suggest that P-O fit creates conditions for autonomy satisfaction by reducing internal conflict between personal values and organizational expectations. When employees need not suppress or compartmentalize their values at work, they experience greater autonomy-not merely in task execution, but in fundamental self-expression. Independence ethical climate amplifies this effect by explicitly legitimizing individual moral agency, thereby satisfying the autonomy need at a deeper ethical-identity level. This dual alignment-values and ethical autonomy-creates synergistic effects on meaning in work that exceed either factor alone.

### Direct effects of P-O fit

The strong direct negative effect of P-O fit on turnover intention (*b* = −0.26) aligns with meta-analytic findings (Kristof-Brown et al., 2005) showing that value congruence consistently predicts retention outcomes across contexts. This effect size is notably stronger than typical correlations reported in Western samples (*ρ* = −0.18; Verquer et al., 2003), suggesting that in Poland’s tight labor market, value alignment may carry heightened importance for retention decisions. When alternative employment options are abundant, employees may prioritize value fit as a key differentiator between organizations.

### Meaning in work as mediator

The mediating role of meaning in work, while statistically significant, demonstrated modest effect sizes (indirect effect ranging from −0.105 to −0.143 depending on ethical climate level). This pattern suggests that meaning in work is one of multiple pathways through which P-O fit influences turnover, rather than the primary mechanism. The relatively small indirect effects align with recent meta-analytic work (Allan et al., 2019) showing that while meaningful work consistently predicts positive outcomes, its effects are often modest compared to more proximal work attitudes like job satisfaction or organizational commitment.

Interestingly, the indirect effect was stronger at higher levels of independence ethical climate, supporting the theoretical proposition that ethical autonomy enhances the meaning-making process. This finding extends previous work on job autonomy and meaning (Hackman and Oldham, 1980) by demonstrating that ethical autonomy-freedom to apply personal moral standards-functions similarly to task autonomy in fostering meaningful work experiences.

### Moderating role of independence ethical climate

The significant moderation by independence ethical climate represents a novel contribution to the ethical climate literature. Previous research has primarily examined how person-climate fit predicts outcomes (Ambrose et al., 2008), but has not investigated whether specific climate types potentiate the effects of P-O value fit. The current findings demonstrate that independence climate functions as a resource amplifier-when present, it strengthens the conversion of value congruence into experienced meaningfulness.

This finding has important theoretical implications for JD-R theory (Bakker and Demerouti, 2017). It suggests that personal resources (value congruence) and contextual resources (ethical climate) interact synergistically rather than additively. Organizations that combine value-based recruitment with independence ethical climates create conditions where personal and contextual resources mutually reinforce each other, producing stronger effects on psychological states and behavioral intentions.

### Theoretical implications

This study advances theoretical understanding in several ways. First, it establishes meaning in work as a psychological mechanism linking P-O fit to turnover intention, extending beyond previously studied mediators like job satisfaction and work engagement. This contribution aligns with calls to integrate positive psychology constructs into organizational retention research (Spreitzer and Cameron, 2012).

Second, the study demonstrates that ethical climate dimensions can moderate value congruence effects, suggesting that organizational ethical cultures shape how value fit manifests in employee experiences. This finding extends both P-O fit theory and ethical climate theory by revealing their interactive effects.

Third, by conducting this research in Poland, the study provides evidence that P-O fit mechanisms generalize to Central European contexts, despite cultural differences in individualism and power distance. This cross-cultural replication strengthens confidence in the universality of P-O fit theory while highlighting potential cultural variations in effect magnitudes.

Fourth, the integration of P-O fit theory, JD-R theory, self-determination theory, and social identity theory within a single model demonstrates how multiple theoretical perspectives can be synthesized to explain complex retention phenomena. This multi-theoretical approach provides a more comprehensive understanding than any single framework alone.

### Practical implications

The practical implications of this study focus on the need for organizations to implement measures to further align their values with those of their employees, to facilitate purpose in work, and to shape an independent ethical climate. Two pertinent recommendations are made. The first recommendation is to devise a recruitment strategy that illuminates the values systems of candidates and how they match or deviate from the organizations’ preferred values. Employees whose values align with their organization’s ones are ideal because they will more easily find meaning in their work and will be less likely to leave the organization.

Organizations without a value codex should consider developing one; and companies that already have one should communicate their preferred values transparently in their internal and external employer branding to give candidates an opportunity to respond to job offers more knowingly, to better identify with the organization, and to learn attitudes that are consistent with the organization’s expectations. Specifically, organizations should incorporate values assessment into structured interviews, provide realistic job previews that authentically represent organizational culture and values, allowing candidates to self-select based on fit rather than presenting idealized portrayals, use assessment centers or situational judgment tests that present value-laden scenarios, observing how candidates’ responses align with organizational values, involve multiple organizational members in selection processes to assess value fit from diverse perspectives.

Company managers, including human resource managers, need to implement ethical standards rooted in the ethical codex of their employees and to emphasize employees’ freedom to make ethical decisions based on their own ethical rules and norms. Practical strategies include replacing rigid ethical rules with principles-based guidance that respects individual moral reasoning while establishing organizational boundaries, training managers to support employee autonomy in ethical decision-making rather than imposing top-down ethical directives, creating psychologically safe spaces for ethical dialog where employees can discuss moral concerns without fear of reprisal, avoiding purely compliance-based ethics training in favor of approaches that develop ethical reasoning capacity and moral imagination. The final recommendation is for organizations to conduct staff training and workshops on finding meaning in work.

### Limitations

This study has some limitations that are mainly connected to the research sample. First, the generalizability of the results is limited by the exclusively Polish population, who were rather homogeneous and mostly religious, and by the large majority of young women with relatively little work experience. The overrepresentation of young, female, early-career employees in the sample may limit generalizability in several ways. Research suggests that women and men may experience P-O fit and meaning in work differently due to socialized gender roles and differing work-life priorities (Kossek et al., 2017). The predominantly female sample may not adequately represent how these mechanisms operate among male employees. The relationship between P-O fit and turnover may be stronger among early-career employees still establishing their occupational identities. Poland’s high religious homogeneity (approximately 92% Catholic) (Główny Urząd Statystyczny [GUS], 2022) may amplify the importance of independence ethical climate, as religiously-oriented individuals often value moral autonomy. Results may differ in more secular or religiously diverse populations.

The sample’s demographic composition—predominantly female (80.3%), relatively young (*M* = 28.64 years), and early-career (*M* = 7.88 years work experience)—warrants careful consideration regarding interpretation and generalizability. Research suggests potential gender differences in how P-O fit and meaning operate.

The relatively young, early-career sample may demonstrate stronger P-O fit effects than would be observed among mid- or late-career employees. Younger workers are still forming occupational identities and may be more sensitive to value (mis)alignment as they explore career paths consistent with their self-concept. Additionally, early-career employees typically have fewer financial obligations and thus greater career mobility, potentially amplifying how meaning and value fit influence turnover cognitions.

Conversely, older employees with established careers, family responsibilities, and accumulated tenure may demonstrate different patterns. They might exhibit stronger organizational commitment that buffers against turnover despite value misfit, or alternatively, they might prioritize meaning more highly given greater awareness of mortality and legacy concerns.

### Future research directions

These demographic patterns necessitate future research examining potential moderating effects of gender and career stage. Multi-group analyses comparing how the proposed mechanisms operate across demographic segments would illuminate boundary conditions and enable more precise theoretical specification. Additionally, research in male-dominated industries or among older workers would test whether our findings generalize beyond young, female, early-career contexts.

This implies the need to conduct studies with more diverse samples, especially those that include different religious denominations or more secular individuals, and that represent various cultural backgrounds (Country Comparison, 2024). Poland differs in power distance and individualism from other countries, and, these cultural dimensions moderate the relationship between P–O fit and turnover intention (Oh et al., 2014).

Future research in more collectivistic countries with smaller power distances could yield interesting results. It should be examined whether these mechanisms operate differently across age cohorts, particularly among Generation Z, Millennials, Generation X, and Baby Boomers. It would be worth to investigate gender differences in how P-O fit translates into meaning and retention outcomes. Next research should not only consider employee–organization adjustment but also present a wider perspective on P–E fit phenomena using other indicators, such as fitness to the job, the supervisor, and the team (Kristof, 1996). Person-supervisor fit and person-team fit may interact with P-O fit in predicting meaning and turnover. For example, misfit with an immediate supervisor might attenuate the protective effects of organizational value congruence.

Second, a longitudinal study should confirm the identified mechanisms from a cause-and-effect perspective rather than merely indicate the direction between variables, as this research did. Future research could use objective measures of organizational values through content analysis of official documents, observation of reward systems, or third-party ratings.

Finally, alternative research models were not tested, including those with different configurations of variables, such as the mediating role of P–O fit in the link between meaning in work and turnover intention.

## Conclusion

Despite these limitations, this study makes important contributions by identifying meaning in work as a mediator and independence ethical climate as a moderator in the P-O fit to turnover intention relationship. Addressing the limitations through future research will refine theoretical understanding, establish causal mechanisms more definitively, identify boundary conditions more precisely, and develop more effective evidence-based practices for enhancing employee retention in diverse organizational and cultural contexts. The convergence of evidence across diverse methodologies, samples, and contexts will ultimately determine the robustness and generalizability of the mechanisms identified in this initial investigation.

## Data Availability

The raw data supporting the conclusions of this article will be made available by the authors, without undue reservation.
